# Two Immigrants with Tuberculosis of the Ear, Nose, and Throat Region with Skull Base and Cranial Nerve Involvement

**DOI:** 10.1155/2011/675807

**Published:** 2011-04-07

**Authors:** Renate A. Richardus, Jeroen C. Jansen, Stefan C. A. Steens, Sandra M. Arend

**Affiliations:** ^1^Department of Infectious Diseases, Leiden University Medical Center, P.O. Box 9600, 2300 RC Leiden, The Netherlands; ^2^Department of Ear, Nose and Throat Diseases, Leiden University Medical Center, 2300 RC Leiden, The Netherlands; ^3^Department of Radiology, Leiden University Medical Center, Leiden, 2300 RC, The Netherlands

## Abstract

We report two immigrants with tuberculosis of the skull base and a review of the literature. A Somalian man presented with bilateral otitis media, hearing loss, and facial and abducens palsy. Imaging showed involvement of both mastoid and petrous bones, extending via the skull base to the nasopharynx, suggesting tuberculosis which was confirmed by characteristic histology and positive auramine staining, while Ziehl-Neelsen staining and PCR were negative. A Sudanese man presented with torticollis and deviation of the uvula due to paresis of N. IX and XI. Imaging showed a retropharyngeal abscess and lysis of the clivus. Histology, acid-fast staining, and PCR were negative. Both patients had a positive Quantiferon TB Gold in-tube result and improved rapidly after empiric treatment for tuberculosis. Cultures eventually yielded *M. tuberculosis*. These unusual cases exemplify the many faces of tuberculosis and the importance to include tuberculosis in the differential diagnosis of unexplained problems.

## 1. Introduction

Tuberculosis (TB) remains a major health problem in the developing world, where almost yearly over 8 million new cases of TB occur [1]. In comparison, in most industrialized countries the incidence of TB is considerably lower, and the majority of TB cases are found among immigrants originating from high-endemic areas. With improvement in economic and social conditions and the use of effective antituberculosis therapy, most high-income countries have initially enjoyed a decline of TB rates during the last several decades. However, with the upsurge of HIV, drug resistance, and the influx of immigrants in, for example, Western-Europe, the USA and Australia, the incidence of TB has not decreased further. It therefore remains important for clinicians to be able to recognize TB, but TB can manifest in virtually every organ or tissue, and complaints are often nonspecific, resulting in delay of diagnosis or worse. Pulmonary TB is the most common clinical presentation, while 15–20% of cases manifest as extrapulmonary or disseminated TB. The most frequent sites of extrapulmonary TB are lymph nodes (48.9%), pleura (25.5%), skeleton (22.7%), genitourinary tract (5.7%), and meninges (5%) [2], but TB can also manifest in the eye, brain, pericardium, peritoneum, abdominal organs, skin, and other sites. In low-endemic areas, individual physicians may see very few, if any TB patients during their career depending on their specialization area. This report describes two patients with rare localizations of extrapulmonary TB in the ear, nose, and throat (ENT) area with skull base and cranial nerve involvement, followed by a review of the literature. These cases illustrate the lack of specific symptoms and signs, the importance of including TB in the differential diagnosis, and the need of an aggressive diagnostic approach of suspected TB in unusual locations.

## 2. Case Report A

**Figure 1:**
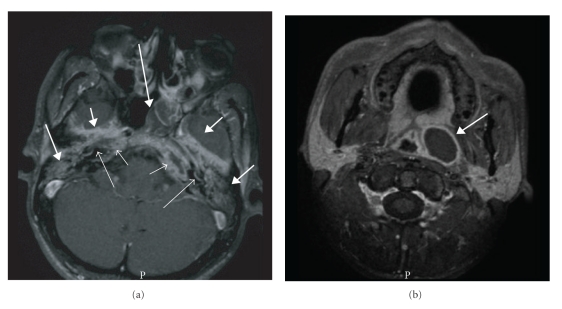
(Case A) Axial T1-weighted MR image with fat suppression after administration of Gadolinium at the level of the petrous bones (a) and just below the skull base (b). Indicated in a are the bilateral inflammatory changes in the petrous apices (short open arrows), bilateral involvement of the basal turn of the cochlea (long open arrows), extensive bilateral pachymeningeal involvement at the temporal lobes (short closed arrows), opacification of both mastoids and middle ears (medium closed arrows), and the mass in the left middle nasal passage extending up to the left Eustachian tube as visualized during fiber endoscopy (long closed arrow). Extension of the abscess into the parapharyngeal space, resulting in a bulge of the contour of the nasopharynx, is also shown (b, medium closed arrow).

A 27-year-old Somalian man, who had immigrated to the Netherlands in 1998, was referred in March 2005 with persistent bilateral otitis media, progressive hearing loss, and tinnitus since eight months, which had been unresponsive to several courses of antibiotics. Other complaints were malaise, fever, anorexia, and weight loss for two weeks accompanied by dizziness and unsteady gait, one week later followed by diplopia and drooping mouth. His sister had been diagnosed with TB during screening on immigration in 1998. At that time, the patient's chest radiography showed abnormalities consistent with past healed TB infection. On physical examination, a malnourished man was seen with complete deafness of both ears. Neurological examination confirmed a right facial palsy and left abducens palsy. Otoscopy showed an extensive polypous inflammatory mass in both external ear canals. During fiber endoscopy a similar mass with purulence was seen in the left middle nasal passage extending up to the left Eustachian tube. The remainder of the examination was unremarkable. Laboratory investigation showed a hemoglobin level of 7.2 mmol/L after hydration, an ESR of 56 mm/first h, and mild elevation of serum transaminases. HIV serology was negative. Tone audiometry showed symmetric mixed hearing loss of 80 to 120 dB. On chest radiography, fibrosis in the apex of the left inferior lobe was seen, unchanged compared to 1998. An MRI showed an inflammatory process of both mastoid and petrous bones with abscess formation on the left extending downwards into the parapharyngeal space resulting in a bulge of the contour of the nasopharynx (Figure 1). An extensive bilateral temporal pachymeningitis was observed as well as bilateral involvement of the inner ear. A low-grade infectious disease process such as TB was suggested. Awaiting further test results, empirical treatment with corticosteroids was started. Histology of biopsies from the external ear canals as well as from the mass extending from the nasopharynx and skull base to the nose showed necrotizing granulomatous inflammation with giant cells. Auramine staining was positive with one acid-fast rod in the nasopharyngeal biopsy, and Ziehl-Neelsen staining and PCR of all biopsies were negative. The Quantiferon TB Gold in-tube assay (Cellestis, Carnegie, Australia), performed three days after starting prednisone, was positive (>10 IU/mL interferon-*γ*, cut-off value 0.35 IU/mL). Hence, the patient was diagnosed with tuberculous otitis media with petrositis extending via the skull base to the nasopharynx. Treatment with four antituberculous drugs (rifampicin, isoniazide, pyrazinamide, and ethambutol) and pyridoxine was initiated. Within several weeks there was clinical improvement with functional recovery of the nervus facialis and abducens. However, severe hearing loss persisted (>90 dB in both ears at all frequencies), requiring a hearing aid. Culture of the nasopharyngeal biopsy yielded susceptible *M. tuberculosis. * Isoniazide and rifampin were continued for a total duration of 12 months.

## 3. Case Report B

**Figure 2:**
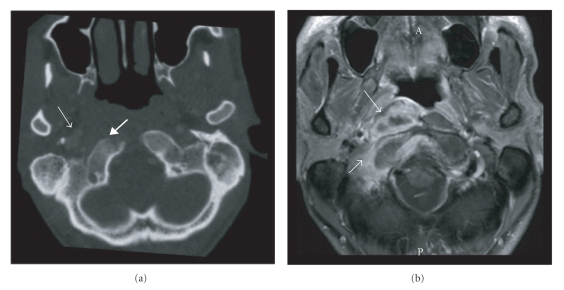
(Case B) Axial contrast-enhanced CT image in bone window (a) and axial T1-weighted MR image with fat suppression after administration of Gadolinium (b) at the level of the foramen magnum. Indicated in (a) are the mass on the right side of the nasopharynx (medium open arrow) and lysis of the clivus (medium closed arrow). The abscess in the retropharyngeal space and prevertebral muscle is better appreciated on MR (b, medium open arrow); edema in the clivus and occipital bone as well as paravertebral soft tissues are also indicated (short open arrow).

In 2005, a 35-y-old man from Sudan, who resided as a refugee in the Netherlands since 3 years, presented with pain in the neck since several months, a sore throat, problems with swallowing, and torticollis to the right. He reported weight loss of five kg in two months. Three months earlier he had been analyzed for unproductive cough, but chest radiography was without abnormalities, and no specific diagnosis was made. On physical examination an ill, transpiring man without a fever was seen. There was a repositionable torticollis to the right shoulder and deviation of the uvula to the right due to paresis of the right N. IX and N. XI. On nasendoscopy, an asymmetric mass with a glazed aspect was seen on the right side of the nasopharynx. The remainder of the examination and the routine laboratorium examination were unremarkable. HIV serology was negative. The Quantiferon TB Gold in-tube assay was positive (>10 IU/mL interferon-*γ*; cut-off value 0.35 IU/mL). Chest radiography showed no abnormalities. CT and MRI showed abscess formation and surrounding edema in the retropharyngeal space and prevertebral muscles on the right, accompanied by usurpation of the clivus and C0-C1 joint (Figure 2). The differential diagnosis included TB. Biopsies of the nasopharyngeal mass obtained during nasendoscopy were not diagnostic. Next, a CT-guided biopsy of the prevertebral mass was obtained under complete anesthesia, showing necrotic material with chronic active inflammation. Auramine and Ziehl-Neelsen staining and PCR for *M. tuberculosis *were negative. Based on the positive Quantiferon test result in the absence of an alternative diagnosis, treatment with four anti-TB drugs was started followed by rapid clinical and radiological improvement with recovery of cranial nerve function. The culture yielded fully susceptible *M. tuberculosis. * Isoniazide and rifampicin were continued for a total of 12 months. At the end of treatment, only partial destruction of the right C0-C1 joint persisted.

## 4. Discussion

Both patients presented with neurological symptoms caused by TB in the ENT region with skull base and cranial nerve involvement, representing a rare manifestation of TB. In a series of 323 cases of extrapulmonary TB, 23.2% presented as ENT localization [3] of which 94.1% in cervical lymph nodes, 4.33% in the larynx, 0.62% in the tonsil, 0.31% in the oral cavity, 0.31% in the middle ear, and 0.31% in the nose. Two smaller studies from India reported roughly similar findings, but included rare cases of TB of the cervical spine, parotid, temporomandibular joint and a retropharyngeal abscess [4, 5]. Mancusi et al. reported that 0.2–1.3% of skeletal TB is localized in the skull, with involvement of the skull base occurring in only a few cases [6]. Thus, our two patients with otitis media, retropharyngeal abscess, and skull base TB represent extremely rare forms of extrapulmonary TB. 

The pathogenesis of ENT TB is thought to result either from primary infection of Waldeyer's ring following transmission of infectious droplets expectorated by a patient with smear-positive TB or by hematogenous spread from a TB focus in the lung or elsewhere. Another possibility is direct inoculation from an endogenous pulmonary TB focus to the larynx, oral cavity, or nasopharynx, yet many patients with ENT TB, including those described in this paper, have no signs of active pulmonary TB at the time of diagnosis. The clinical manifestations of ENT TB may be caused either by a mass effect of the inflammatory process or by destruction of anatomical structures, both of which often occur simultaneously in TB. Due to the complex anatomy of the ENT area, with the skull base, cranial nerves, and C0-C1 and atlantoaxial joints all in close proximity, TB in this area can present with manifestations of different systems as was also the case in our two patients. 

That isolated skull base TB is extremely rare may be explained by the fact that bone TB has a predilection for large and weight-baring bones such as the vertebrae and large joints, while the skull base is a nonweightbaring bone with limited articulation. In patient A, direct spread from a focus of prolonged untreated tuberculous otitis media seems most plausible, as the otogenic symptoms preceded the cranial nerve dysfunction by eight months suggesting intracranial spread from lateral to medial. In patient B spread starting in a primary site in the nasopharyngeal lymphoid tissue and hematogenous spread to the skull base were both possible. The unproductive cough for which the patient was analyzed before the cervical symptoms arose and for which no specific cause was found would be compatible with involvement of N. X at the skull base or spread from a nasopharyngeal localization. 

The pathogenesis of tuberculous otitis media (TOM) may be by spread via the Eustachian tube from the retropharyngeal lymphoid tissue, hematogenously from another TB focus, or through direct implantation through the external auditory canal in a patient with tympanic membrane perforation [7, 8]. TOM occurs in less than 0.9% of chronic suppurative otitis media cases and is often not associated with pulmonary TB while clinical signs are variable and nondistinctive [8], leading to delayed diagnosis. Symptoms that may suggest TOM are (painless) otorrhea, conductive hearing loss, facial palsy, (multiple) tympanic perforations, granulation of middle ear mucosa, and soft tissue invasion or bone necrosis on CT-scan [7–10]. It is important to consider TOM in the case of chronic otorrhea not responding to topical antibiotics, although the use of aminoglycoside ear drops can mask TOM. Even when TB is considered, cultures may remain negative because of the overall low sensitivity of biopsies of limited size containing a low bacillary burden or through interference by aminoglycoside ear drops. PCR can be used, but has not been validated in this setting. Of 52 patients with TOM in South Korea [8], only 9.6% had a history of TB, 5.8% reported contact with a patient with active TB, and 9.6% had signs of (old) TB on their chest radiography. Patient A had a positive contact history and a chest radiography consistent with past healed TB. In that series, the interval between initial symptoms and diagnosis varied from less than a year to more than ten years, illustrating the chronic course and diagnostic delay due to noncharacteristic presentation. The main symptom was otorrhea with mucopurulent discharge, 13.5% reported otalgia, 28.8% tinnitus, and 13.5% experienced dizziness, probably as a result of destruction of the semicircular canals. Peripheral facial palsy was found in 9.6%, which is higher than that among patients with otitis media in general, thus raising suspicion of TOM, especially in the absence of cholesteatoma. Facial palsy was associated with a shorter duration of symptoms (mean 9.4 mo. versus 106.3 mo.). Conductive hearing loss occurred in 56.6% and a mixed hearing loss in the remainder. Otoscopic examination revealed perforations of the tympanic membrane or adhesions. Temporal petrositis may present with the triad of otorrhoea, pain in the distribution of the trigeminal nerve, and abducens nerve palsy, which is classically known as Gradinego's syndrome [11]. 

Primary skull base TB is extremely rare, most likely resulting from hematogenous spread from the lungs, transmission from pharyngeal lymphoid tissues, or by extension from the mastoids or middle ear, paranasal sinuses, or the convexity. There are only few case reports of TB of the clivus [6, 12–15], with or without retropharyngeal abscess, as in patient B. Presentation with headache and unilateral foramen jugulare syndrome (paralysis of the ninth, tenth, and eleventh cranial nerves) has been reported [12]. Patient B presented with loss of the N. IX and N. XI function and possibly involvement of N. X manifesting as nonproductive cough without abnormalities on chest radiography. Skull base tuberculoma can mimic a malignant tumor, underscoring the essential value of histology. Involvement of the skull base secondary to TOM has, rarely, been described in case reports [11, 16, 17], which may be the result of direct extension through bone destruction or by way of venous channels. Simultaneous pyogenic and TB infection can occur which can mask the TB component. 

Similar to patient B, several other patients have been reported with retropharyngeal tubercular abscess secondary to involvement of the cervical vertebrae through direct extension [4, 18]. In children, it may result from involvement of the retropharyngeal lymph nodes [5], but not in adults as these structures generally disappear after 4-5 years of age [18]. Symptoms of retropharyngeal abscess are dysphagia, odynophagia, hoarseness, bulging of the posterior pharyngeal wall, swelling in the neck, and airway obstruction. Abscess formation due to extension from spondylitis is initially contained behind the prevertebral fascia and presents as a retropharyngeal abscess or, through lateral extension, as a sternomastoid abscess or parotid mass. 

In our two patients, radiological findings played an important diagnostic role. CT is reportedly the best imaging technique for TOM [7]. Soft tissue attenuation in the entire middle ear cavity, preservation of the mastoid air cells without sclerotic change, and soft tissue extension to, or mucosal thickening of, the external auditory canal were more frequent in patients with TOM compared to those having pyogenic chronic otitis with or without cholesteatoma. Erosion of the ossicles and scutum was more frequent in TOM. Temporal bone CT scans of 23 South Korean patients with TOM showed bone destruction that involved the cortex of either the external auditory canal or the outer cortex of the mastoid bone in 26.1% of cases [8]. More marked bone destruction involving the skull base as well as the mastoid bone was found in only one patient (4.3%). Cholesteatoma occurs most frequently in the Prussak's space as opposed to the cortical erosion caused by TOM. Diffuse temporal bone destruction should prompt to include TOM in the differential diagnosis. 

When direct diagnostic methods for TB may take several weeks and have limited sensitivity, indirect methods can be used to either support or invalidate the suspicion of TB. The value of the tuberculin skin test in persons who have been vaccinated with BCG is limited due to problems with interpretation of a positive test result. Interferon-gamma release assays (IGRAs), such as Quantiferon TB-Gold, are in vitro immunodiagnostic tests that measure effector T-cell-mediated response to *M*. *tuberculosis*-specific antigens [19]. These tests are not affected by BCG vaccination which is a major advantage over the tuberculin skin test. IGRAs were superior to the tuberculin skin test in active TB [20], although the sensitivity of IGRA for active TB is incomplete [21]. A recent study found that higher quantitative interferon gamma values were associated with active TB [22]. In both our patients, the result of the Quantiferon TB-Gold assay was strongly positive, which confirmed the suspicion of TB, justifying the initiation of treatment after biopsies that were obtained. However, IGRA cannot differentiate between past latent TB infection and active TB. Thus, it remains of utmost importance to obtain direct proof through histology, staining, PCR, and/or culture. If adequate facilities for imaging, targeted biopsy, and microbiological diagnostics are not available and TB is strongly considered, it may be justified to treat a patient empirically for TB and evaluate carefully for a clinical response, on condition that it is recognized that clinical deterioration not necessarily invalidates the diagnosis TB, but may indicate a paradoxical response to treatment as occurs frequently during TB treatment [23].

## 5. Conclusion

These two cases of rare manifestations of TB, with skull base and cranial nerve involvement, illustrate the importance to include TB in the differential diagnosis of unexplained clinical problems. Clues that should prompt the clinician to consider skull base TB are cranial nerve dysfunction, radiographic finding of a mass, destructive process, or diffuse temporal bone destruction in the absence of another explanation. TOM should be considered in case of chronic otorrhea especially if occurring in an individual originating from a TB-endemic region.
